# “Let’s talk about money”: how do poor older people finance their healthcare in rural Ghana? A qualitative study

**DOI:** 10.1186/s12939-019-0927-0

**Published:** 2019-03-20

**Authors:** Williams Agyemang-Duah, Charles Peprah, Prince Peprah

**Affiliations:** 1 Department of Planning, Kwame Nkrumah University of Science and Technology, Kumasi, Ghana; 2 Department of Geography and Rural Development, Kwame Nkrumah University of Science and Technology, Kumasi, Ghana; 3 Built and Natural Environment, Sheffield Hallam University, Sheffield, UK

**Keywords:** Healthcare finance, Poor older people, Rural Ghana, Qualitative study

## Abstract

**Background:**

Older people utilise more healthcare services and are likely to incur higher healthcare expenditure, however, data on their healthcare financing mechanisms are scarce in low-and middle- income countries including Ghana. In this study, we aimed at exploring how poor older people finance their healthcare in rural Ghana.

**Methods:**

We conducted in-depth interviews and focus group discussions with 60 study participants comprising 30 poor older people, 15 healthcare providers and 15 caregivers in Atwima Nwabiagya District of Ghana. Data were analysed using thematic analytical framework and presented based on an a *posteriori inductive* reduction approach.

**Results:**

The study revealed that poor older people finance their healthcare through personal income, family support, Livelihood Empowerment Against Poverty grants and National Health Insurance Scheme subscription. It was also found that poor older people spent between GH¢ 20 and 250 on drugs, laboratory test and hospitalisation anytime they access a healthcare facility.

**Conclusion:**

The findings contribute to our understanding of how poor older people finance their healthcare in rural Ghana. We argue that health stakeholders should strengthen healthcare financing mechanisms for poor older people for optimal healthcare use.

## Introduction

Discussions on sustainable healthcare financing mechanisms for vulnerable people are still ongoing [1]. Ghana has implemented a number of healthcare financing mechanisms since independence as a way of ensuring financial sustainability [2]. After independence, Ghana’s healthcare system was financed by tax revenue and external assistance without any charges at the point of service [1]. This was due to the political ideology of promoting free healthcare and education in the country [3]. However, this healthcare financing mechanism was not able to be sustain because of the country’s poor economic performance [3] and consequently, user fee was implemented in the healthcare system in 1980s [1]. That notwithstanding, user fee system became a key barrier to healthcare accessibility and utilisation mainly because of poverty especially in the rural areas [1].

Due to the challenges that were inherited in the user fee system popularly known as “cash and carry”, the National Health Insurance Scheme (NHIS) was introduced in 2003 through an Act of Parliament (Act 650, Amended Act 852) and full implementation started in 2004. The NHIS remains a major healthcare financing policy for people in Ghana including older people. To improve financial accessibility for the poorest and most vulnerable, there is a free membership of the NHIS to certain portions of the population—exempt group. This group consists of Social Security and National Insurance Trust (SSNIT) pensioners, people above 70 years, pregnant women, children under 18 and paupers [4]. It covers about nineteen in twenty of morbidity burden situations such as outpatient services, inpatient services, deliveries, diagnostics, medicines and all emergencies [5]. These diseases include malaria, diarrhoea, respiratory tract infections, skin diseases, hypertension, diabetes, asthma, breast and cervical cancers. It however excludes diseases such as HIV and AIDS, brain and heart surgeries, among others. Other treatments exempted from the NHIS package include childhood immunisations, tuberculosis and mental healthcare. Meanwhile, medicines used in mental health may be used in general practice as well and such drugs are listed on the NHIS [6].

The NHIS financing arrangements are made in such a way that purchasing contracts are signed between the NHIS district offices and the accredited healthcare providers. The details of the contract include the prescriptions of general drugs for the treatment of particular health conditions. The healthcare practitioners therefore submit claims for services provided to the NHIS insured clients to the NHIS district offices for reimbursement [7]. Yet, challenges such as inadequate funding sources and insufficient premium are associated with the NHIS [8]. A study has revealed that after rendering health care services, medical providers were not repaid with the funds that were required to restock their centers, purchase equipment, and hire the professionals required to ensure quality healthcare delivery [9]. The operational and financial sustainability of health insurance interventions serve as barriers to NHIS in Africa including Ghana [10].

Given this background, understanding of sustainable sources of financing healthcare across the globe has become a major discussion among policy actors and stakeholders [8]. At the same time, research on sources of healthcare financing among older people has gained an important root in healthcare arena because of the increasing ageing population. For instance, the global ageing population as at 2015 was 901 million which is predicted to hit 2.1 billion by 2050 [11]. Also, Ghanaian older people aged 65 or over represent 4.6% of the entire population [12], and it is estimated to be 14.1% by 2050 [13]. Due to high exposure to communicable and non-chronic communicable diseases among older people [14, 15], they tend to utilise more healthcare and as a consequence, are likely to have higher expenditure on healthcare use.

Studies have reported that family support [16–19], Livelihood Empowerment Against Poverty grants and rents on property are the major sources of funds available to older people. Although, there is a consensus to achieving a universal health system [20], studies on individuals’ sources of healthcare financing particularly among vulnerable older people remain unavailable in low-and middle-income countries including Ghana. In order to ensure a sustainable and sound healthcare financing for poor older people in Ghana, there is the need to know their available healthcare financing sources, amount spent on healthcare and items they spend their income on when they visit a healthcare facility. In relation to this, this study, sheds light on a category of vulnerable population that are at risk of catastrophic health expenditures in rural Ghana. This study would therefore help to strengthen healthcare financing mechanisms for poor older people for optimal healthcare use in Ghana and also go a long way to contribute partly to the realisation of the United Nations health-related Sustainable Development Goals.

## Methods

### Study setting and design

This study was extracted from a wider Ageing, Health, Lifestyle and Health Services Survey (AHLHS) conducted between 1 and 20 June 2018 in the Atwima Nwabiagya District of Ghana. Other aspects of the original research have been reported elsewhere [21]. A retrospective cross sectional design with mixed methods strand of study was employed in the wider study. Data from this study drew on reports and findings from one of the qualitative sections of the original study exploring how poor older people finance their healthcare in rural Ghana. The qualitative design helped to a gain better understanding because it provided an avenue for the study participants to openly share their experiences, perspectives and views on the topic [22, 23]. It further enabled the authors to adopt interpretivist paradigm and subjectivist epistemology [24] where original feelings, experiences and belief systems of the study participants to be of much prominence. Aside from this, the interviewee and the interviewer were able to interact which generated a meaningful collaborative effect [25]. The use of this approach further enabled the authors and the study participants to explore new knowledge areas that could have been difficult to capture in a quantitative study.

The researchers purposively chose three rural communities comprising Kobeng, Amadum-Adankwame, and Offinso Adagya and five formal healthcare facilities consisting of Nkawie Toase Government Hospital, Afari Community Hospital, Akropong Health Centre, Dr. Frimpong -Boateng Medical Centre, and Mount Sinai Hospital in Atwima Nwabiagya District, of Ghana. These five healthcare facilities were selected for the study because they were the first five facilities poor older people mostly access and utilise in the study area. Of the five healthcare facilities visited, three (Afari Community Hospital, Dr. Frimpong -Boateng Medical Centre and Mount Sinai Hospital) health care facilities were privately owned but they all accepted the national health insurance card. The other two (Akropong Health Centre and Nkawie Toase Government Hospital) healthcare facilities are owned by the government.

### Sampling and recruitment procedures

Purposive and convenience sampling techniques were employed to choose 30 poor older people, 15 caregivers and 15 formal healthcare providers making 60 study participants for the study. Poor older people were considered to be persons aged 65 years or above receiving financial support from the Livelihood Empowerment Against Poverty programme (LEAP) [21]. The LEAP is a financial transfer policy sponsored by the Government of Ghana, the World Bank and the United Nations International Children's Emergency Fund (UNICEF). It provides financial protection to households and persons in Ghana that are extremely poor so as to reduce their poverty level [26, 27], as well as free enrollment on the NHIS [26]. Beneficiaries include older people who are 65 years or above, persons with disability and orphaned and vulnerable children [26, 28]. It also attempts to improve the livelihood and healthcare use of the beneficiaries in order to better the socio-economic development of the country [29]. The beneficiaries receive a bi-monthly minimum and maximum amount of GH¢ 64 and 106 (13.42USD- 22.23 USD as at the period of the study) respectively. The individual decides on how to use the money either on healthcare or other basic needs. Involvement of older people under the LEAP programme helped the authors to identify those categorised as poor older people in the study area.

Family members or any other persons providing care for poor older people were considered to be caregivers. On the other hand, formal healthcare providers were those health professionals such as doctors, nurses, and midwives who had knowledge about the diagnosis or treatment of diseases afflicting people and who had worked in the medical field for at least 3 years preceding the study [21]. The respective sample size allocation for the study participants was where a point of saturation was reached. At this point, the authors were not getting new information. Hence, the selection of the study participants did not consider the study population in the study area [30]. Poor older people and caregivers were selected using convenience sampling technique because of their availability and readiness to take part in the study [31–33]. Formal healthcare practitioners were purposively selected due to their in-depth knowledge on the topic [23, 34], and as such could provide information to enrich the study [23].

### Data collection instrument and procedure

In-depth interviews and focus group discussions (FGDs) were conducted for data. In a qualitative study of this nature, in-depth interviews and FGDs are mostly recommended [22, 23, 35, 36]. Three FGDs and 60 in-depth interviews were conducted for the study data. The interviews provided room for interviewees to express their opinions and beliefs with regard to the subject under investigation [33]. In situations where further explanations were needed, the study participants were probed and this enabled the authors to get the required information. [33]. Poor older persons, healthcare providers and caregivers were all participated in the interviews. The same questions were given to the poor older persons and caregivers. The interview guide was divided into sections based on the objective of the study. The first section focused on the background data comprising gender, education, religion ethnicity, age, enrollment on the NHIS and whether the participants are active enrollees of the NHIS. The second section focused on healthcare expenditure, sources of funds for healthcare and items poor older people spend their income on when they access a healthcare facility. Whereas interviews with poor older people and caregivers were conducted at their homes which were free from interference from any third party, that of the healthcare providers were conducted at a free consulting room after the end of their usual daily activities. These places provided a more friendly and relaxed atmosphere for both the interviewer and the interviewee. This was due to the fact that the place was devoid of fear and suspicion [37]. The interviews were supported with informal and personal conversations conducted by the first author who had in-depth knowledge in medical geography, health policy, and health development. Interviews with the participants lasted approximately 60 min.

On the other hand, FDGs were conducted at convenient places such as school and church building that were free from noise and interferences. The discussion lasted 60 min and ended at a point of saturation. In the FGDs, the conversation was done by someone who was more knowledgeable about the issue under investigation to ensure a better understanding of the issue by the study participants [34]. The moderator’s function was to facilitate the group discussion rather than lead the discussion [23]. The FDGs were basically focused on how poor older people finance their healthcare. The interviews and FDGs with poor older people and caregivers were conducted in ‘Twi’ (the local dialect of the study participants), whiles discussions with health providers were carried out in English. Interviews and FGDs were audio-recorded with an informed consent from the study participants and handwritten field notes were made [23].

### Trustworthiness

With the use of suitable sampling techniques, the authors were able to enhance credibility, transferability, conformability and dependability which enhanced trustworthiness in this study. Member check also helped to ensure the credibility of the study results. Summaries of the results were shared among those involved in the study and the participants affirming that the findings reflect their expressed views, feelings, and experiences. Moreover, the duration for the interaction with the study participants was lengthy with both the interviews and FGDs lasting approximately 60 min. Also, the transcripts, recorded interview, and notes were given to an expert in qualitative research for critical scrutiny to ensure quality control.

### Ethics approval and consent to participate

The Committee on Human Research Publication and Ethics (CHRPE), School of Medical Sciences, Kwame Nkrumah University of Science and Technology and Komfo Anokye Teaching Hospital, Kumasi, Ghana provided ethical clearance for the study (Ref: CHRPE/AP/311/18). Informed written and verbal consents were obtained from the study participants before data were collected. The study participants were also assured of strict confidentiality and anonymity of the data they provided.

### Data analysis

All interviews and FDGs that were conducted in Twi were translated into English. Translations were cross-checked with the audio records and handwritten field notes to ensure validity, reliability and quality control. All authors read the transcripts and handwritten field notes for general understanding. We performed back-to-back translation on selected transcripts in order to check the accuracy of the translations. The data coding then started until theoretical saturation was reached and an a posteriori inductive method applied [38]. This approach helped the authors to develop broad and consistent themes and enabled them to derive true-to-life experiences after engaging with the study participants, instead of relying on their prior, possibly biased, knowledge of the issue under investigation. The authors then read and reviewed the transcripts and interview notes several times, and topics were extrapolated and compared to the responses for the purpose of identifying similar trends and differences. The essence of the thematic analysis was that it helped to identify, analyse and report patterns within data, and also aided in organising and describing the data in rich detail [39]. The study results were presented based on themes, and the normative views of the study participants were presented using quotes and excerpts.

## Results

### Background characteristics of participants

The study participants who were involved in the in-depth interviews were 60 with 30 being poor older people (users), 15 formal healthcare providers and 15 caregivers. In respect of the formal healthcare providers, 9 were females and 6 were males. All the formal healthcare providers (15) had attained tertiary level of education. Twelve of the formal healthcare providers were Christians. Also, 12 of the formal healthcare providers were of Akan ethnicity. With regard to the caregivers, all of them were females (15), 8 had no level of education, 14 were Christians and 13 were of Akan ethnicity. With regard to poor older people, 23 were females and 7 males demonstrating a higher enrollment of females on the LEAP programme than males in the study area. Due to the gendered nature of poverty among females and the need to reduce poverty among them, about 75% of the LEAP beneficiaries in Ghana and for that matter the study area are females. Also, it could be seen that users were comparatively illiterate in that a total of 19 had no education at all. The preponderance of the users were Christians (27) whereas 25 were of Akan ethnicity. Again, 9 of the users were aged between 65 and 69 years. Furthermore, all the poor older people had enrolled on the NHIS before but 7 had not renewed their NHIS card because they did not know that the card must be renewed annually. Also, long travel time and cost to the NHIS renewal centres as well as poor quality healthcare for insured participants were other factors cited by the poor older people for not renewing their NHIS card. (Table 1).

**Table 1:** Sample characteristics of the study participants

		Category of Respondents (*N* = 60)		
Variable	Category	Users (poor older people) (*N* = 30)	Providers (*N* = 15)	Caregiver (*N* = 15)
Gender	Male	7	6	–
	Female	23	9	15
Education	None	19	–	8
	Basic	8	–	5
	Secondary	3	–	2
	Tertiary	–	15	–
Religion	Christianity	27	12	14
	Islam	3	3	1
Ethnicity	Akan	25	12	13
	Northerner	5	3	2
Age (years)	65–69	9	–	–
	70–74	6	–	–
	75–79	3	–	–
	80–84	4	–	–
	85–89	3	–	–
	90 or over	5		
Have you registered for NHIS before?	Yes	30	–	–
	No	–	–	–
Is your NHIS active?	Yes	23	–	–
	No	7		
If No, why?	Do not know it must be renewed annually	3		
	Travel time and cost is too high	1		
	Poor quality of care for those paying with NHIS	2		
	Poor network	1		

### Summary of main and sub-themes

In all, three main and eight interlinking sub-themes were respectively identified based on the analysis of the accounts of the study participants. The views of the study participants on healthcare financing mechanisms among poor older people in Atwima Nwabiagya District of Ghana were the main constructed categories from the data. (Table 2).

**Table 2:** Key themes and associated sub-themes

Main themes	Sub-themes
Sources of funds for healthcare	• *Family*
	• LEAP grant
	• *National Health Insurance Subscription*
	• *Personal Income*
Healthcare expenditure	
	• Between GH¢ 20 and GH¢ 250 (4.1945 USD- 52.4307USD)
Healthcare items	
	• *Drugs* • Laboratory test • *Hospitalization*

NB: 1 USD = GH¢ 4.76820 as at of the time of the survey

### Healthcare expenditure

Most of the study participants indicated that poor older people spent between GH¢ 20 and 250 when they access healthcare services. This was indicated in the quotations below from poor older people, caregivers and healthcare providers.

For instance, one poor older person from Kobeng expressed:

*“I was at hospital for about 2 months. I paid about GH¢ 200. My son paid for the bills at the hospital*” (Poor older person, interview).

A different poor older person from Offinso Adagya stated:

“*The last time I was taken to the hospital, I paid GH¢100*” (Poor older person,, interview)

Another poor older person from Amadum - Adankwame indicated:

“*I incurred about GH¢ 40*” (Poor older person, interview)

A poor older person from Offinso Adagya further stated:

“*I have paid about GH¢250. Initially I was paying for all the bills but a time came that my children have to continue from there*” (Poor older person, FGDs).

The views from healthcare providers and caregivers were in line with what the poor older persons said regarding their healthcare expenditure. For example, one caregiver from Offinso Adagya expressed:

*“The last time I took my mother to the hospital, she paid GH¢ 90”* (*Caregiver*, interview).

A healthcare provider from Mount Sinai Hospital indicated:

“*On the average older people spend less than 260 cedis here anytime they access health. Some even pay GH¢ 40 especially those health insurance while those with no health insurance pays at times GH¢ 120. Under extreme case some can pay more than GH¢ 150”* (Health care provider, interview).

Another healthcare provider from at Akropong Health Centre mentioned:

*“Here healthcare is affordable for older people. Most of the older people have insurance so they pay less. Older people spend between GH¢20 and 100 especially those with insurance”* (Healthcare provider, interview).

### Sources of financing of healthcare

The study revealed four main sources of healthcare financing for poor older people in the study area. These included personal income, family, LEAP grants and NHIS subscription.

#### Family

Most of the study participants indicated that family members have been instrumental in paying the medical expenses or expenditure of poor older persons in rural Ghana. For instance, the poor older people mentioned that their children have been financing their healthcare. The quotations below reinforce the views of poor older people. One poor older person from Offinso Adagya commented:

*“My children pay for my health bills”* (Poor older person, interview).

A provider from Nkawie Toase Government Hospital mentioned:

*“Older people get money from their relatives to pay their medical expenses”* (Healthcare provider, interview).

Similarly, a poor older individual also expressed:

*“My children pay for my health bills. Even as I speak, I cannot go to the hospital alone. My grandchild has to accompany me to the hospital*” (Poor older person, FGDs)

The above quotation indicated that apart from family members paying the medical bill of their poor older people, they also accompanied them to the healthcare facility to seek for treatment when the need arises. This shows the role of family members particularly wards in the healthcare financing and utilisation of poor older people in the study area.

The financial support of family members especially wards in the healthcare use of poor older people was necessary because of poor economic activities among poor older people in the study area. This was due to the fact that most of them did not engage in any economic activity which could have enabled them to earn income to pay their medical bills expenses. A poor older person from Kobeng explained:

“*My children pay for all my health bills. At my age, I cannot work. I solely rely on their help”* (Poor older person, FGDs).

A caregiver from Amaduma-Adankwame summed up the above discussion:

“*The children of poor older persons support them in paying their medical bills because you see at their age, they don’t work so they don’t have money to pay for their bills*” (Caregivers, interview).

#### Personal income through donation

One source of healthcare financing identified from the study participants was personal income. Most of the poor older people indicated that they relied on their own source of income to cater for their healthcare expenditure. Some reasoned that at times they get money from people in the community for healthcare. However, it was made known that this source of income was not regular which affects their frequency of healthcare. For instance, one poor older people from Kobeng responded:

*“I pay for all my health bills. There is no one to help. This makes me not to attend to hospital regularly”* (Poor older person, interview).

A caregiver from Amaduma -Adankwame said:

*“Poor older people rely on their own source of money to finance their health care” (*Caregiver, interview).

A healthcare provider from Afari Community Hospital mentioned:

*“Even though poor older persons are enrolled on the NHIS, the NHIS does not cover all the medications so they have to do top up at times with their own money*” (Healthcare provider, interview).

#### LEAP grant

It was made known that the LEAP funds remain one of the sources of healthcare financing for poor older people. Most of the participants also stated that there was a free NHIS registration for beneficiaries of the LEAP programme in Ghana. The rationale was to help remove financial barrier which prevents access to quality health services and also protects poor people especially older persons from the impoverishing effects of medical expenses. However, the use of LEAP grants was left for the beneficiaries to decide. To this end, recipients may choose to spend the money on food, healthcare or other basic needs. Even though it was acknowledged that users spent some of the LEAP grants on their health care, the study participants claimed that the amount was too small as it ranges from GH¢ 64 to 106 (13.42USD- 22.23 USD as at the period of the study). This, they said, was relatively small to cater for most of their healthcare needs even though they were enrolled on the NHIS. They further argued that not all of the drugs were covered under the NHIS. Besides, most of the participants expressed that not all their medical conditions were covered under the NHIS. Participants added that diseases such as dialysis for chronic kidney failure, heart and brain surgery, appliances and prosthesis including optical aids, hearing aids, orthoptics (diagnosis and treatment of defective eye movements and coordination) among others were not covered under the NHIS. Hence, there was the need to pay for these treatments anytime they visit a healthcare facility. The participants further noted that part of the LEAP grants was used to cater for other healthcare needs such as hospitalisation and laboratory test.

That notwithstanding, most of the study participants indicated that the LEAP grants had been supporting poor older persons to cover their medical expenses. Evidently, a poor older person from Kobeng said this:

*“The source of funds to healthcare is the LEAP grants. I rely on the LEAP assistance before I can go to the hospital”* (Poor older person, interview).

A poor older person from Offinso Adagya further commented:

*“The LEAP also supplements my children’s effort”* (Poor older person, FGDs).

A healthcare provider from Mount Sinai Government Hospital stated:

*“I know poor older persons are enrolled in the LEAP programme. The LEAP transfer has been helping them to pay their health care expenses”* (Healthcare provider, interview).

Although, it was agreed that the LEAP grants assisted poor older persons to pay their medical bill, the funds were not released on time and this resulted in borrowing among the recipients. One poor older person from Kobeng said:

“*I rely on the LEAP grant, but it does not come on time. When I am sick, I have to contract loan to seek for health care and later pay it when the LEAP funds are released”* (Poor older person, FGDs).

A key issue raised by poor older person has to do with the limited amount (GH¢ 64 to 106) of the LEAP transfer. This, most of the study participants, stated were insufficient to cater for the basic needs of poor older people including their healthcare.

In light of this, one caregiver from Kobeng mentioned:

“*The LEAP funds support poor older people in seeking health care but I will tell government to make it monthly (payment) or increase the current amount paid to me” (*Caregiver, interview)

#### National Health Insurance Scheme Subscription

All the three groups of the study participants mentioned that all NHIS subscription was a form of healthcare financing mechanism for poor older people. Most of the poor older people were covered under the NHIS. As a result, they had to pay no or little cost depending on the nature of treatment and the drugs prescribed to them. It was noted that not all the drugs are covered under the scheme.

A provider from Afari Community Hospital commented:

*“The insurance covers some of the drugs. There are some drugs that are not cover by the insurance and with this the aged have to do some top up”* (Healthcare provider, interview).

A caregiver from Amaduma -Adankwame said:

“*Most of the poor older people rely on the NHIS to access healthcare*” (Caregiver, interview).

Reinforced by this assertion was a quotation from a poor older person from Kobeng which was stated as follows:

*“Health insurance is one of the source of funding available of healthcare available to the older person”* (Poor older person, FGDs).

Healthcare provider from Nkawie Toase Government Hospital explained:

“They *use national health insurance because they think it is reliable and it is cheaper to enroll on it. Especially with the diabetics, when you come and you are admitted at this ward, the first test the insurance would cover it but with the subsequent ones, the client will have pay*” (Healthcare provider, interview).

One healthcare provider from Akropong Health Centre stated:

*“Some of them have insurance so they are able to afford but it is normally difficult for those without insurance. Even with those with insurance, sometimes they need to top up depending on the various drugs that we prescribe to them*” (Healthcare provider, interview).

As evident in the quotation below, a provider from Dr. Frimpong-Boateng stated:

“*Oh, the health insurance has really helped, if you don’t have it and you sleep here, we charge you GHS20.00 a day, but if you have the health insurance, accommodation is free. Health insurance covers accommodations and some drugs. In the government side, insurance covers lab test but for here lab is cash and carry”* (Healthcare provider, interview).

### Healthcare items

The study found three key healthcare items poor older people spent their income on. These comprised drugs, laboratory test and hospitalisation as explained in the subsequent section.

#### Drugs

The study participants expressed that poor older persons spent their income on drugs anytime they visit a healthcare facility for treatment. It was made known that most poor older persons suffered from non-chronic communicable diseases such as diabetes, high blood pressure and as a result have to consume drugs daily. In this case, the study participants professed that drugs take huge sum of their income especially those that were not covered under the NHIS.

The quotations below from poor older persons from Offinso Adagya and Amaduma -Adankwame respectively explained the above assertions:

“*I use the little money I have to buy drugs from the drug store*” (Poor older person, interview).

*“If I spend money buying drugs. This is because I do not get the prescribed drugs at the hospital. I have to buy the rest outside. Most of them (drugs) I buy outside the hospital are expensive*” (Poor older person-FGDs).

Similarly, healthcare providers from Afari Community Hospital and Akropong Health Centre respectfully expressed:

*“Drugs, lab test and many more. For the diabetics, they spend their income on glucose syrups to monitor their blood sugar”* (Healthcare provider, interview).

*“Review of the NHIS because if you take this facility into consideration, it is a health centre so it means we don’t have most of the drugs” *(Healthcare provider, interview).

These views were also endorsed by caregivers as indicated the quotations: One caregiver from Amadum- Adankwame mentioned:

*“The older persons spend their income on drugs that would sustain them”* (Caregiver, interview).

#### Laboratory test

All the three sets of the study participants mentioned that laboratory test was one of the healthcare items areas poor older people spent their income on. It was made known that poor older people had to pay for the laboratory cost. It was indicated that the laboratory test helped the healthcare providers to know the actual health problems facing poor older people so that they could correctly prescribe drugs to them. It was noted that some poor older people were not able to tell which health problem they were suffering from. As such, the healthcare providers indicated that they had to make them go through series of laboratory tests before they could know their health problems.

The following was a quotation from a poor older person who resided at Amaduma-Adankwame:

“*I go through normal activities such laboratory test. Out of the lab test, the doctor then prescribes the require drugs for me*” (Poor older person, FGDs).

Another poor older person from Offinso Adagya mentioned:

“*At times I go through series of laboratory tests before given the prescribed drugs. With this lap test too, I have to pay money*” (Poor older person Interview).

This was what a poor older person from Kobeng had to say:

“*The nurses will first attend to me after that they will direct me to see the doctor. The doctors then examine me and ask to go to the lab to do test at a cost*” (Poor older person-interview).

A caregiver from Kobeng stated:

“*My grandmother had an accident and was taken to Nkawie Toase Government Hospital. The nurse examined her and was discharged. A day after I saw that her face was swollen. I took her back to the hospital and was asked to go for laboratory test*” (Caregiver, interview).

A healthcare provider from Nkawie Toase Government Hospital mentioned:

“*More to this, some can’t tell what is wrong with them so we allow those people to go to the lab to do test and based on the lab test, we are able to prescribe drugs. But this normally increases their medical bills since the lab goes at a fee*” (Healthcare provider, interview).

Another healthcare provider from Dr. Frimpong - Boateng Medical Centre reckoned:

“*I talked about lab being cash and carry, when you come, you have to go for lab and most of them are not able to pay because they don’t have money*” (Healthcare provider, interview).

#### Hospitalisation

Most of the study participants indicated that poor older persons spent their income on hospitalisation. The study participants mentioned that on serious cases, poor older persons are admitted at the hospital for some days to help them recover from their time before they were discharged. It was revealed that admitting them at the hospital comes at a cost. For instance, a poor older person from Kobeng indicated:

*“I have been admitted at the hospital before. When I was admitted, I needed to pay for admission fee and cost of treatment*” (Poor older person, interview).

A provider from Nkawie Toase Government Hospital did say:

*“In patient when you come and your condition demand that, you will be admitted. Most of them also prefer to be admitted so as to release them of a little pressure and discomfort from home. They believe that when they are admitted here, they get comfort. You also know that outpatient is the first port of contact when you go to any health facility*” (Healthcare provider, interview).

Another healthcare provider from Afari Community Hospital said:

“*Some of them come for inpatient health care especially in severe cases*” (Healthcare provider, interview).

A caregiver from Offinso Adagya said:

“*My grandfather has been admitted at the hospital before and with this, we paid some money to cover admission fee and treatment fee*” (Caregiver, interview).

## Discussion

This study explored healthcare financing mechanisms among poor older people. The objective of the study was in three folds. The first was to understand healthcare expenditure among poor older people. The second was to explore the sources of financing healthcare among poor older people. The last was to investigate healthcare items poor older persons spend their income on when they visit a healthcare facility.

With regard to healthcare expenditure, the study revealed that poor older people spent between GH¢ 20 and 250 anytime they access healthcare. This was relatively similar to the finding of Jacobs et al. [40] who indicated that older people seeking healthcare in Cambodia spent US$36.6 (equivalent to GH¢178.97) on medical care in a month. It has been reported that older people experience a much higher disease burden and are likely to spend more on healthcare in a month. This healthcare cost could be attributed to the complex treatment requirement of older people because they tend to suffer from multiple health challenges [40]. Poor older people are faced with high burden of chronic diseases and this would result in higher healthcare expenditure [41]. Their high burden of diseases, in some cases, results from poor health behaviour practices such as smoking, substance abuse, sedentary lifestyle, inadequate consumption of fruits and vegetables [42]. Therefore, to scale down healthcare expenditure among poor older people, health actors or stakeholders should educate older people on the need to adopt good health behaviour or lifestyle. This would either prevent them from diseases, or reduce their burden of diseases or complications [43], thereby decreasing their healthcare expenditure.

The study revealed multiple sources of funds for healthcare for poor older persons. These included family support, LEAP grants, NHIS subscription and personal income. This finding agreed with earlier studies that indicated support from family members as a major source of funds for older people [16–19, 44]. Social support from family members, friends and community members provided avenues for health information delivery and other assistance in terms of funds for traveling and payment of healthcare utilisation [14]. The finding that family and friends constituted an important source of funds for healthcare of older people is critical as it could inform social policy debate on the need to reshape support mechanisms to ensure the welfare of vulnerable older persons in Ghana [45]. Building and sustaining closer relationship with family members and close friends is capable of providing a buffering effect for health and may increase the likelihood of obtaining informal support through information on healthcare services and also as a way to defray healthcare expenditure in later life [45]. This suggests the need to strengthen family support system through education, advocacy and awareness creation since it plays a key role in healthcare financing in later life. Such education could be done by the Department of Social Welfare in the various Metropolitan, Municipal and District Assemblies across the country. Traditional authorities and the media are also key stakeholders that can contribute partly to this advocacy, awareness creation and education in Ghana.

This study was in line with a research conducted by Anning [17] that LEAP transfer constitutes one of the source of funds for healthcare. It was not a surprised to find most of the study participants indicating the LEAP grants as their source of funds for healthcare because all the poor older people were enrolled on the LEAP programme. It was found that some of the LEAP funds were used to purchase drugs that were not covered under the NHIS. They also used part of the money to cover other healthcare needs such hospitalisation and laboratory test. This demonstrates the role of a social protection programme such as LEAP in contributing towards health care financing among poor older people in rural Ghana. However, the participants indicated that the amount (which ranges from GH¢ 64 to 106) was too small to cater for most of their healthcare needs or demands such as purchases of drugs that were not covered under the NHIS. The poor older people recommended for an upward adjustment of the LEAP grants to enable them meet their healthcare needs. However, due to the qualitative nature of our study, we could not measure how the LEAP has impacted on healthcare financing of the poor older people under the LEAP programme in the study area. Therefore, a quantitative study is needed in this research area to better our understanding in this knowledge area.

The NHIS subscription serving as a source of funds for financing healthcare of older people is consistent with previous studies [14, 46]. More importantly, health insurance in Ghana was introduced to remove the financial barrier to healthcare and to increase healthcare utilisation by eliminating user fees at the point of use [46]. This finding has validated the research output of Agyemang-Duah et al. [21] which found that the NHIS allows poor older people to utilise public healthcare services for little or no cost. In Ghana for instance, the NHIS serves as an important social intervention and welfare strategy by scaling down the healthcare cost for vulnerable older persons [47]. This study however found that seven of the participants had not renewed their NHIS card because they did not know that it must be renewed annually. Besides, long travel time and cost to the NHIS renewal centres, poor quality healthcare for insured participants and network failure (which slows down the issuance of the NHIS card) serve as barriers to renewal of NHIS. Aspect of this finding concurred with the assertion by Duku et al. [7] that people do not enroll on the NHIS as a result of delay which results from queuing for a long time at the healthcare facility. Further, the operational and financial sustainability of health insurance interventions serve as barriers to health insurance interventions in Africa including Ghana [10]. This calls for the attention of policy makers to address barriers associated with the NHIS in Ghana for optimal healthcare utilisation among poor older people.

The study found that drugs, laboratory test and hospitalisation were the major items poor older persons spent their income on anytime they access a healthcare facility. It was revealed that drugs were the first healthcare item the study participants spent their income on. Although most of the study participants were enrolled on the NHIS, they still had to pay for drugs because the NHIS does not cover all medications. Hence, patients had to pay for the cost of the drugs that were not covered under the scheme [5]. The study further revealed that hospitalisation and laboratory test consume the income of the study participants. Hospitalisation has been noted as a determinant of healthcare expenditure [48]. The above healthcare items, therefore, constitute the healthcare demands of poor older people and as such, provide important information for policy makers in providing financial cover for poor older people under the LEAP programme in rural Ghana. It was further suggested that the Ministry of Health, Ghana Health Service and other important health actors should put measures in place to ensure free healthcare for poor older people in Ghana.

The strengths of this study deserve to be remarked on. In fact, this is the first study to have qualitatively explored this important knowledge area in Ghana. The study could contribute to the realisation of the United Nations health-related Sustainable Development Goal. It is also critical to helping policy makers and health stakeholders to formulate policies that aim at strengthening the mechanisms for financial protection for poor older people for optimal healthcare use. In spite of these strengths, following limitations were noteworthy. The interpretation of these findings must be undertaken with caution since the study selected participants by employing non-probability sampling techniques. Due to this same reason, we were not able to estimate the average healthcare expenditure of the study participants. Also, we were not able to establish differences between active and non-active NHIS enrollees as regards their healthcare financing mechanisms. Lastly, since this study is the first of its kind in Ghana, the authors were not able to get enough local and international literature to validate or otherwise findings of this current study. We therefore encourage more works to be done in this knowledge area to contribute to the scanty literature on the topic. Such future studies should endeavour to establish differences between active and non-active NHIS enrollees as regards their healthcare financing mechanism.

## Conclusion

This study explored how poor older people finance their healthcare in rural Ghana. The study reported that participants finance their healthcare from various sources and also incur healthcare expenditure on multiple healthcare items. The findings reported in this study have, therefore, contributed to our understanding of how poor older people finance their healthcare in rural Ghana. The policy implications drew from the findings for the attention of policy makers include provision of free healthcare and strengthening of healthcare financing mechanisms for poor older people in rural Ghana.

## Abbreviations

AHLHS: Ageing, Health, Lifestyle and Health Services Survey
CHRPE: Committee on Human Research Publication and Ethics
FDGs: Focus Group Discussions
LEAP: Livelihood Empowerment against Poverty programme
NHIS: National Health Insurance Scheme
SSNIT: Social Security and National Insurance Trust
UNICEF: United Nations International Children's Emergency Fund
